# Developing sustainable practices to protect children and adolescents from infectious diseases

**DOI:** 10.1002/pdi3.78

**Published:** 2024-06-05

**Authors:** Janne Estill

**Affiliations:** ^1^ Institute of Global Health University of Geneva Geneva Switzerland; ^2^ School of Basic Medical Sciences Lanzhou University Lanzhou China

After the COVID‐19 pandemic, news of infectious disease outbreaks can be alarming. When an unusually large number of respiratory infections in children were reported in North‐East China in 2023, it brought flashbacks from early 2020 when COVID‐19 changed the everyday life from complete normality into an unprecedented emergency situation within just a few weeks. The fact that the observed cases were primarily affecting children was particularly worrisome. Although it became apparent that the situation was caused by previously known pathogens, these outbreaks reminded us that the threat of infectious diseases is not over.

## 1 THE *MYCOPLASMA PNEUMONIAE* OUTBREAKS IN 2023: WHAT ACTUALLY HAPPENED?

In May 2023, an increased incidence of respiratory infections was observed in China, concerning particularly children. However, the situation became more severe during the autumn, with Beijing and the Liaoning province being the epicenters of the outbreak. The elevated incidence was reported by the Chinese National Health Commission on November 13, 2023, while 10 days later, both the Chinese health authorities and the World Health Organization confirmed that the situation was caused by existing pathogens, most notably *Mycoplasma pneumoniae*.^1^ A major contributor was the start of the first cold season since the lifting of the COVID‐19‐related restrictions, causing many seasonal respiratory infections to circulate simultaneously. In parallel, similar outbreaks were also seen in other countries, for example, Russia, the United States, Germany, and Denmark. The situation caught some attention also in the mass media in many countries for a while.

## 2 WHAT CAUSES INFECTIOUS DISEASE OUTBREAKS AND HOW TO CONTROL THEM?

The emergence and spread of infectious diseases depend on a complex network of determinants ranging from the biomedical characteristics of the pathogen to societal structures and human behavior. Sources for new potentially threatening infections are numerous. The ongoing changes in land use and climate increase the interactions between humans and animals, creating opportunities for zoonotic infections to transfer to humans.^2^ Existing viruses and bacteria can mutate, and strains that are fitter—for example, more transmissible or able to escape immunity or present resistance to treatment—take over, causing severe outbreaks of existing diseases. In the fight against new pathogens and variants, surveillance plays a major role. However, relying on routine findings reported by patients and clinicians is not sufficient: when the existence of a potentially hazardous new pathogen is identified, it has likely been already spreading widely for a long time. During the COVID‐19 pandemic, routine surveillance systems, such as wastewater screening^3^ or random testing of travelers^4^ or health facility attendees, were proposed or implemented, but the attention and investments to these interventions has substantially decreased since the end of the pandemic phase. It is crucial to ensure that routine surveillance systems continue to operate, and the tools developed during the pandemic are adapted into long‐term regular use. Early detection of potentially hazardous pathogens and variants can facilitate timely response, planning of mitigation measures, and development of treatments and vaccines.

Although the characteristics of infectious diseases, such as the basic reproduction number *R*_0_, are often presented as features of the pathogen itself, this is only half of the truth. The transmission procedure depends also on the intensity of human contacts, which in turn is associated with many social, cultural, and behavioral factors. During the last pandemic, lockdowns and other heavy restrictions in social contacts were unavoidable, but they also had serious consequences for the mental well‐being of the population, particularly children. Another problem is that in the absence of a comprehensive surveillance system, the need for such measures can be identified only when the disease is already spreading widely. What is therefore needed is lighter behavioral interventions that can be adopted regularly also during normal times, being thus able to decrease the infectiousness of communicable diseases right from the beginning of future outbreaks.

## 3 CHILDREN IN INFECTIOUS DISEASE PANDEMICS

Children are a special population in many ways, and the protection of children is without question a top priority in public health. In the early days of the COVID‐19 pandemic, it was a great relief that the first evidence demonstrated that children are less affected than older age groups. However, this relieving information also led to the controversy that children were often ignored in the planning of the epidemic response. Public health measures involving children were criticized based on the oversimplifying claim that children are not affected by the virus, ignoring issues such as the protection of family members at high risk, the risk of unknown long‐term sequelae, or the eventual need for school closure at short notice in the case of severe outbreaks.^5^

Children have a key role in infectious disease epidemics for many reasons. The immune system of children is not yet fully developed, increasing the vulnerability to many infections. Children who are in the process of learning their social skills are particularly disadvantaged by any strong restrictions in societal life. Moreover, children's contact networks differ also essentially from those of adults. Most children living in the same neighborhood attend the same school, while adults tend to have more scattered contact networks through their workplaces and social contacts. Children therefore act as the link that connects the families living in the same area: infections brought by adults from elsewhere can rapidly spread in the local community through schools. Children are also essentially the only age group who can be reached through a single public institution—schools—which offers an ideal platform for disseminating knowledge and organizing health interventions.

## 4 PREPARING FOR FUTURE PANDEMICS: HOW TO PROTECT CHILDREN AND THE ENTIRE SOCIETY?

Should we be worried about a future pediatric‐driven pandemic? There is certainly no need to panic, but new infectious disease outbreaks and pandemics will come, so it is wise to prepare in advance. Instead of considering pandemics as events that ultimately get resolved and can be forgotten, we should rather keep learning from the past and build better response systems that can timely detect and act on any future threats from new infectious agents. Children and adolescents are in many ways in a central position—they have the most to lose, but also possess a great potential for action. Infectious disease epidemiology is a fascinating field of science that combines mathematics, biology, and social sciences—so why not integrate it into the education of children? Children are fast in learning, understanding, and can be proud to contribute to public health interventions, showing that even small children can take actions that have a great impact on the entire society. Sudden long‐term closures of schools can have serious consequences for the development of children—but it would not be harmful even in normal circumstances to take better care of ventilation and spend more time outdoors; develop hybrid learning methods so that children with an acute respiratory infection can stay a few days at home without dropping out; and integrate the necessary infrastructure for vaccinations and mass testing into schools. With such sustainable interventions, we can be better prepared for future epidemics and ensure that children can continue to have as normal a life as possible even in times of public health emergencies.

## AUTHOR CONTRIBUTIONS

**Janne Estill**: Conceptualization, literature search and writing the manuscript.

## CONFLICT OF INTEREST STATEMENT

The author serves as Deputy Editor‐in‐Chief of Pediatric Discovery. To minimaze bias, he was excluded from all the decision‐making related to the acceptance for publishing. The author declares no other conflict of interest.

## ETHICS STATEMENT

Not applicable.

## CONSENT TO PARTICIPATE

Not applicable.

## Data Availability

Data sharing is not applicable to this article as no datasets were generated or analyzed during the current study.

## References

[1] World Health Organization (WHO) . Disease Outbreak News: Upsurge of Respiratory Illnesses Among Children – Northern China. World Health Organization. Accessed November 23, 2023. https://www.who.int/emergencies/disease‐outbreak‐news/item/2023‐DON494

[2] The Lancet Infectious Diseases . Twin threats: climate change and zoonoses. Lancet Infect Dis. 2023;23(1):1. 36502819 10.1016/S1473-3099(22)00817-9

[3] Jahn K , Dreifuss D , Topolsky I , et al. Early detection and surveillance of SARS‐CoV‐2 genomic variants in wastewater using COJAC. Nat Microbiol. 2022;7(8):1151‐1160. 35851854 10.1038/s41564-022-01185-x PMC9352586

[4] European Centre for Disease Control and Prevention (ECDC) . Pilot Study Outline for Targeted Genomic Surveillance of SARS‐CoV‐2 in Travellers in Response to a Worsening or Unknown Epidemiological Situation in a Third Country. ECDC. Accessed January 13, 2023. https://www.ecdc.europa.eu/sites/default/files/documents/covid‐19‐pilot‐study‐targeted‐genomic‐surveillance‐sars‐cov‐2‐in‐travellers.pdf

[5] Keiser O , Agoritsas T , Althaus CL , et al. A public health strategy for SARS‐CoV‐2, grounded in science, should guide Swiss schools through the coming winter. Swiss Med Wkly. 2021;151:w30086. 34652090 10.4414/smw.2021.w30086

